# Community pharmacist counselling practices in the Bisha health directorate, Saudi Arabia –simulated patient visits

**DOI:** 10.1186/s12913-020-05554-2

**Published:** 2020-08-13

**Authors:** Hassan Al Qarni, Tahani Alrahbini, Ayidh M AlQarni, Abdullah Alqarni

**Affiliations:** 1 Sabt Al Alaya Hospital in Bisha Health Directorate, Ministry of Health, 7501 riyadh - Al Naseem Dist, Bisha, 67841–2152 Saudi Arabia; 2 Department of pharmacy and allied science, Riyadh Elm University, 8728 King Fahd Branch Road - Al Namudhajiyah Dist, Riyadh, 12734–5077 Saudi Arabia

**Keywords:** Community pharmacy, Pharmacist, Counselling, Patient simulation, Visit time, Duration of counselling

## Abstract

**Background:**

Many organizations have suggested a minimum standard regarding pharmaceutical counselling for patients, which includes information about the name of the drug, the description the drug, the indication for use, how to use it, the method of treatment, precautions, adverse reactions, and any contraindications. Correspondingly, the World Health Organization (WHO) has recommended that pharmacists spend at least 3 min with each patient to provide counselling. This study aimed to evaluate the counselling practices of community pharmacists for both over-the-counter (OTC) and prescription-only medicines in the Bisha Health Directorate, Saudi Arabia.

**Method:**

This was a cross-sectional study designed to evaluate simulated patient visits to observe real counselling practices of community pharmacies in the Bisha area; 73 pharmacies were visited. Two scenarios were used in this study. The first scenario was for a prescription-only medicine, and the second scenario was for an OTC medicine. The duration of counselling was recorded for every visit.

**Results:**

A total of 105 visits to the 73 pharmacies were conducted under the following scenarios: scenario 1 = 73 visits and scenario 2 = 32 visits. The average time for the simulation was 110 s for scenario 1 and 73.4 s for scenario 2. There was a statistically significant negative correlation between the duration of counselling and patient simulation visits that occurred in the morning (Spearman’s rho = − 0.396, *p* = 0.001).

**Conclusion:**

It was shown that patient counselling needs to be given greater emphasis in community pharmacies. In this study, the time spent for patient counselling failed to meet the minimum WHO standard. Thus, pharmacists must be encouraged to spend at least 3 min on patient counselling.

## Background

The Bisha Health Directorate, which is part of Asir Province, is considered to be a rural area. In 2007, the population was 199,613 people, and there were only 25 community pharmacies [1]. By 2017, the population had increased to 388,055 people, and the number of community pharmacies had increased dramatically to 125 [2]. From these data, it can be seen that the population increased by approximately 94% over this 10-year period, with an approximately 500% increase in the number of community pharmacies during the same period. Therefore, the pharmacy business is growing rapidly in the Bisha area with no or limited information on how pharmacies can provide good pharmacy practice (GPP), especially counselling, to the community.

Pharmacists hold a position of trust among the community. According to the laws and regulations in Saudi Arabia, medicines distributed in community pharmacies, i.e., private community pharmacies, are divided into two classes [3]. The first class includes prescription-only medicines, which are defined as medicines that cannot be dispensed without an authorized prescription. The second class includes non-prescription or over-the-counter (OTC) medicines, which are defined as medicines that can be supplied without a prescription. OTCs are considered to be safe and effective if they are used according to the guidelines available on the package or the label [4]. In addition, the World Health Organization (WHO) has recommended that pharmacists spend at least 3 min with every patient for orientation and counselling [5]. However, in many developing countries, they spent less than 3 min with patients. For example, the average time reported in Cyprus is 149 s; in Brazil, 53.9 s; in Sudan, 21.8 s; in Nepal, 86.1 s; in Tanzania, 77.8 s; in Nigeria, 12.5 s; and in Bangladesh, 23 s [5, 6].

One of the essential services of a community pharmacy is patient counselling, for both prescription-only and OTC medicines. Many organizations have provided guidelines and recommendations to pharmacists about patient education and counselling for both prescription-only and OTC medicines. Pharmacists should provide the following information to their patients: name of the drug, description of the drug, indication, how to use it, method of treatment, precautions, adverse drug reactions, and any contraindications [7–10]. In addition, all the guidelines emphasize the importance of gathering enough information from the patient to ensure patient safety, patient understanding about the medication, and whether the medication will meet patient needs [7–9, 11]. Okumura et al. (2014) found in their systematic review study that patient counselling can improve drug and disease knowledge, clinical outcomes, patient satisfaction, and quality of life of patients. Moreover, by law, pharmacists are not allowed to dispense any medicine without a prescription, written by an authorized person, unless the medicine is categorized as OTC [3].

Toklu et al. (2010) focused on implementing GPP, which includes counselling, in a specific district in Istanbul, Turkey. They found that GPP was poorly applied in community pharmacies [12]. Additionally, Gokcekus et al. (2012) conducted a similar study in the northern Turkish region of Cyprus. They concluded that GPP in that region was insufficient and needed improvement [6]. Halial et al. (2015) studied the evidence-based practice of community pharmacists for OTC medicines in Parana State, Brazil. They concluded that there was a major problem with poor knowledge regarding evidence-based practice among community pharmacists, which negatively affected counselling practices [13]. Tully et al. (2011) studied the predictors of no counselling, no questioning, and no information provided to patients during counselling in community pharmacies in Sweden. Their conclusions suggested the importance of therapeutic classes and busy times as predictors of no counselling about prescription medicines in Swedish pharmacies [14].

Counselling, which is part of GPP, has been investigated during the past years in Saudi Arabia, especially from the legal perspective of prescribing medicines. Many studies have been conducted on the misuse of antibiotics in community pharmacies. Patient counselling was included in the evaluation process in some of these studies. Many studies from 1992 until now described the level of counselling, especially patient counselling, and the extent to which Saudi regulations were followed. Three studies found that community pharmacies did not adhere to pharmaceutical law and regulations [15–17]. A systematic review in 2016 concluded that the dominant practice of the community pharmacies in Saudi Arabia was to dispense medicines without providing any pharmaceutical care to the patients [18].

There is limited information about pharmacy counselling practices in the Bisha Health Directorate. Therefore, this study aimed to evaluate the counselling practices of community pharmacists for both OTC and prescription-only medicines in the Bisha Health Directorate, Saudi Arabia.

## Methods

### Study design

This study was a cross-sectional study of simulated patient visits to community pharmacies; two scenarios were evaluated. This study was developed to observe the real counselling practices of community pharmacists.

### Inclusion and exclusion criteria

The study included any private pharmacy, i.e., community pharmacy, in the Bisha Health Directorate. Government pharmacies and any community pharmacy that was still closed after two visits were excluded from this study.

### Sample selection

The investigators went to any community pharmacy that was open for business; the aim was to visit 90 of the 125 pharmacies in Bisha. The power of the analysis was estimated to be 90% confidence with a 10% α level interval. The Bisha Health Directorate is divided into three areas, which are the centre (Bisha city and its close villages), east (Tathleeth and its close villages), and west parts (Sabt-Alalaya and its close villages); all areas were included in this study. The original plan for this study was to reach 30 pharmacies in each area. It was However, if the number of pharmacies was not achieved in one area, then additional pharmacies from another area were visited to compensate. It took 3 months to complete the simulated patient visits with both scenarios (from August 2017 to October 2017).

### Assessment method

#### Simulated patient visit

The simulated patient visits were used to assess the counselling services provided by pharmacists in community pharmacies. This assessment method was derived from a pharmacy practice-based research study conducted in Saudi Arabia in 2015 [19]. The simulated patients were volunteer pharmacists who were trained to perform specific scenarios to evaluate the counselling provided by the community pharmacists [20]. The simulated patient was expected to become familiar with the standard information of the counselling process and the laws and regulations in Saudi Arabia because at the end of every visit, they were required to complete a checklist of counselling received. The participating pharmacists performed role plays before starting their visits and collecting data to ensure that the scenarios were suitable and compatible with real cases. If the volunteered pharmacist recognised by community pharmacist as a pharmacist, e.g. Colleagues, friend etc., then the visit will not be included in the study. In the scenarios, there were drug-disease interactions and drug allergies to be aware of. Additionally, to avoid bias, the participants were requested to use simple Arabic language, to avoid any jargon and to not provide any further information that could affect the results.

The first scenario was related to prescription-only medicines (amoxicillin + clavulanic acid), whereas the second scenario focused on OTC medicines (ibuprofen). A second pharmacist, acting as an observer outside the pharmacy, recorded the time (in seconds) of the discussion between the simulated patient and the pharmacist.

### Statistical analysis

Quantitative data were analysed using the Statistical Package for Social Science (IBM SPSS) version 22 for Windows. Categorical data were expressed as frequencies and percentages. Comparisons between groups were performed using the Mann-Whitney test, and correlations were performed using Spearman’s rho. A *p* value of < 0.05 indicated statistical significance.

## Results

**Fig. 1:**
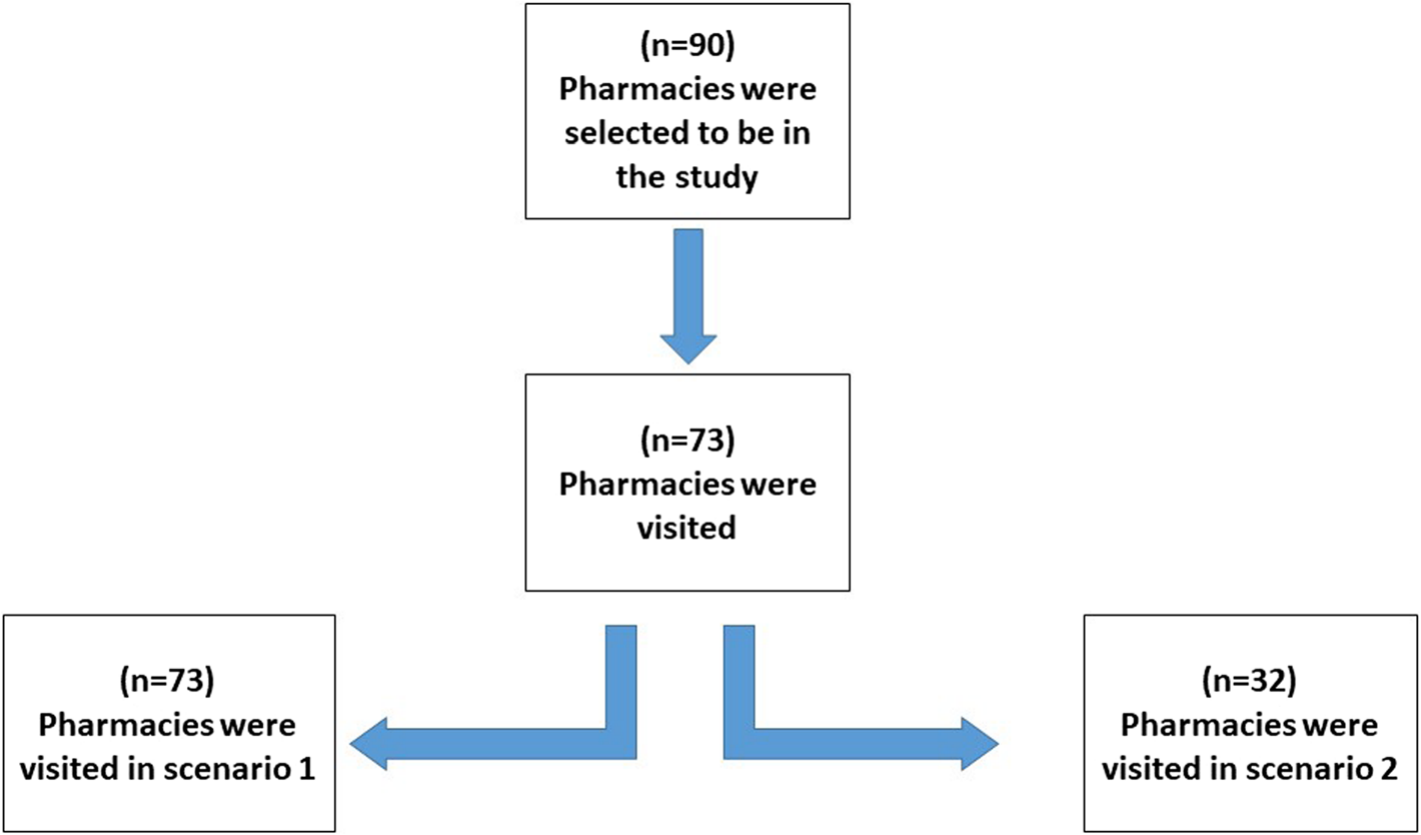
Flowchart of the number of pharmacies visited in both scenarios

**Fig. 2:**
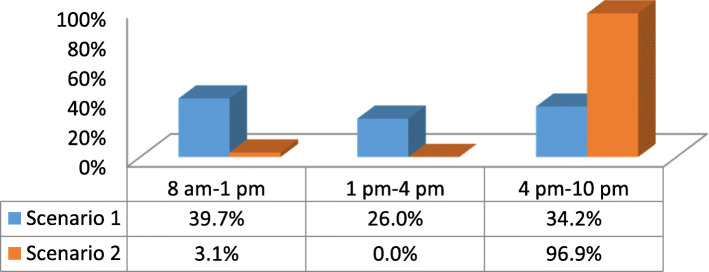
Time of day of simulation patient visits

A total of 73 of 125 pharmacies in the Bisha area were visited. Almost all pharmacies that were opened at that time were visited. There were 17 closed pharmacies not included in this study. Each closed pharmacy was visited twice before they were excluded, and it was not clear if these pharmacies were permanently closed or or they were closed for other reasons. The total number of visits to the community pharmacies was 105. Scenario 1 had 73 visits, and scenario 2 had 32 visits (see Fig. 1). Figure 2 shows the time of day of the simulation visits for both scenarios 1 and 2. The data regarding the presence and content of counselling during medication dispensing, the types of questions asked, and the information measured were collected on the checklist and reported in Table 1. The majority of pharmacists did not ask about previous use of the requested medications (4% for scenario 1 and 14% for scenario 2) or any history of drug allergy (2% for scenario 1 and 0% for scenario 2). Moreover, pharmacists were not aware of the recommended patient counselling practices or whether the patient had any concerns regarding their medication. No pharmacist offered any written information about the medication. Information on dose was the most common type of information provided during the visits. The mean (± SD) times for the simulations for scenarios 1 and 2 were 110 (± 54.8) seconds and 73.4 (± 35.4) seconds, respectively. A total of 27.4% of the pharmacists were able to detect potential drug allergy side effects in scenario 1, whereas no pharmacist was able to detect the problem in scenario 2. Additionally, there was a statistically significant inverse correlation between the duration of counselling and the time of day of the simulation visit (rho = − 0.396, *p* = 0.001).

**Table 1:** Description of the counselling received by simulated patients requesting medication in the two scenarios

		Number answering yes (percent)	
		Scenario 1 (*n* = 73)	Scenario 2 (*n* = 32)
Counselling before patient demanded information	Asked questions	51 (69.9)	31 (96.9)
	Provided information	28 (38.4)	3 (9.4)
Counselling after patient demanded information	Asked questions	36 (49.3)	17 (56.7)
	Provided information	20 (27.4)	17 (53.1)
Questions asked	Asked for prescription	11 (15.1)	3 (9.4)
	Asked who the medicine was for	46 (63.0)	0 (0.0)
	Asked whether the patient had taken this medicine before	4 (5.5)	15 (46.9)
	Asked if other medicines were currently being taken	0 (0.0)	0 (0.0)
	Asked if allergic to any medicine	2 (2.7)	0 (0.0)
	Asked if there were any questions or concerns about this medicine	0 (0.0)	0 (0.0)
Information provided	Name of the medicine	38 (52.0)	0 (0.0)
	Dose	47 (64.4)	16 (50.0)
	How to take the medication (e.g., before or after meals)	24 (32.9)	25 (78.1)
	Duration of use	6 (8.2)	5 (15.6)
	Possible adverse drug reactions, warnings, and precautions	0 (0.0)	0 (0.0)
	Identified drug-drug, drug-disease, drug allergy, or side effects from the scenario	20 (27.4)	0 (0.0)
	Was a choice of products offered?	23 (31.5)	6 (18.8)
	Was any written information offered?	0 (0.0)	5 (15.6)
Time for the simulation (in seconds)	Mean	110.0	73.4
	SD	54.8	35.4
	SEM	6.4	6.3
	Minimum	40.0	30
	Maximum	320	165

Mann-Whitney tests showed a statistically significant relationship between the duration of counselling for the simulation and the drug-drug interactions, drug-food interactions, and side effects identified during the scenario (*p* = 0.000). No significant relationship was found between the time taken for the simulation and whether a choice between different products was offered (*p* = 0.472). However, a statistically significant relationship was found between the time taken for the simulation and whether the pharmacist asked for the prescription (*p* = 0.000).

## Discussion

The present study focused on counselling practices in community pharmacies in the Bisha Health Directorate in Saudi Arabia. The simulated patient visits showed that approximately 50% of pharmacists informed patients about the dose of the medication. Additionally, 8 and 15% explained the duration of use to the patients in scenario 1 and scenario 2, respectively. However, none of the pharmacists explained the possible side effects to the simulated patients. Almost 27% of the participants in scenario 1 provided information about drug interactions, and 15% in scenario 2 provided written information. The actual average time for the simulation was 110 s.

Many organizations have recommended pharmacists spending sufficient time with each patient for orientation and counselling. In the present study, the investigators focused on the World Health Organization’s (WHO) suggestion to spend at least 3 min, which is 180 s, with each patient [4]. However, the present study showed that the pharmacists spent less than 2 min, i.e., 110 s, with each patient. By comparing the observed counselling times from other international studies, the findings in the present study were better than those obtained from Brazilian, Suadanian, Nepalese, Tanzanian, Nigerian and Bangladeshian studies [5, 6]. However, the Cyprus study showed that pharmacists spent more time, i.e., 149 s, dispensing medication, than the those in the present study [6]. Even though the 110 s observed in the present study was higher than that in other studies, this is still not enough to provide each patient with proper counselling. The reason why the pharmacists did not spend enough time with each patient is not known. However, it could be said that the counselling practices were suboptimal. The pharmacists might not be used to asking about the patients’ current medications, medical history, or any other issue that are required for counselling. Additionally, pharmacists might not have proper knowledge about GPP [21, 22]. Also, the duration of counselling was likely to be shorter in the afternoon as pharmacies are busier then. Since, there was a statistically negative correlation between the duration of counselling and the time of day of the simulation visit. The Spearman’s rho was − 0.396 with a *p* = 0.001 [14]., Furthermore, there was a statistically significant relationship between the time of the simulation and the mention of drug-drug interactions, drug-food interactions, and side effects during scenario 1 (*p* = 0.000). If the pharmacist spends more time with a patient, then they can provide better patient counselling, which has the benefit of optimizing drug use and the quality of life of the patient [6, 22].

Only 11% of the pharmacists requested the prescription from patients. In previous Saudi studies, it was confirmed that prescribed medicines were dispensed without a prescription [16, 17, 19, 21]. Even though the simulated patient informed the pharmacist that he had been advised by a friend to use a particular medication to treat his condition, this did not concern the pharmacist. This point has two perspectives. The first is the legal viewpoint. According to pharmaceutical laws and regulations, antibiotics should not be supplied without an authorized prescription, which is to be presented and kept in the pharmacy [16, 21, 23]. However, in the present study, the dominant practice in almost all community pharmacies was to dispensing products without asking for a prescription. The second perspective is that not all pharmacists were Saudi citizens, and they may not have graduated from Saudi universities. Therefore, they might lack knowledge regarding the rules and regulations of pharmacy practice in Saudi Arabia. Additionally, they may have been less concerned about prescription-only medicines and OTC medicine regulations and counselling.

Almost 30% of the pharmacists in scenario 1 were able to detect the potential patient harm, which was that the patient could be allergic to penicillin. Furthermore, only 31.5% of the pharmacists were able to provide alternative treatment or transfer the patient to a physician. One possible explanation for this is that the pharmacists, who did not detect the potential risk had a lack of evidence-based knowledge regarding medicines and how to search for information. Another study also highlighted this issue of imperfect knowledge among pharmacists in Brazil [13].

The limitations of the present study must be considered. The original sample size for the study was 90 pharmacies in the Bisha Health Directorate. However, when we searched for the pharmacies, it was found that many pharmacies had been recently closed or were not open at that time of visiting. Therefore, the final sample size was only 73 pharmacies. The resources and time available were also limitations. For example, sometimes it was difficult to find the pharmacy because there was no address map or GPS location, and we had to travel more than 200 km to look for the pharmacies. It was also difficult to find pharmacists to participate in this study, with only two pharmacists and one health professional volunteering to travel and help with the simulation patient visits. This issue affected most scenario 2, which has a lower number of visits than scenario 1.

## Conclusion

This study showed that the patient counselling provided by pharmacists in private community pharmacies in the Bisha area was suboptimal. A greater emphasis must be placed on pharmacists’ patient counselling in all community pharmacies. Additionally, the time spent on patient counselling was below the minimum WHO standard; thus, community pharmacists must be encouraged to spend at least 3 min per patient on counselling. The stakeholders should promote an intensive training programme for the pharmacists to educate them about counselling practice in Saudi Arabia. Furthermore, the private pharmaceutical sector requires a proper collaborative plan to provide and deliver their main job, which includes providing the proper effective counselling to the patient. Further studies of patient counselling practices among community pharmacies are need; in particular, studies are needed to address the reason why the pharmacists did not spend enough time with each patient, which is not yet well known.

## Data Availability

The datasets generated and analysed during the current study are available from the corresponding author on reasonable request.

## Abbreviations

WHO: World Health Organization
OTC: Over the counter medication
GPP: Good pharmacy practice
IBM SPSS: The Statistical Package for Social Science
SD: Standard deviation
IRB: Institutional review board
